# Immune profiling shows limited systemic changes in children with autism after autologous cord blood transfusion

**DOI:** 10.3389/fimmu.2026.1738417

**Published:** 2026-07-01

**Authors:** Yik-Lam Cho, Zoe Mei Yun Tay, Nicholas Kim Huat Khoo, Rachel Meau Yenn Cheong, Su Li Poh, Judith Ju Ming Wong, Katherine Nay Yaung, Martin Wasser, Salvatore Albani, Joyce Ching Mei Lam, Chui Mae Wong, Joo Guan Yeo

**Affiliations:** 1 Translational Immunology Institute, SingHealth Duke-NUS Academic Medical Centre, Singapore, Singapore; 2 Paediatrics Academic Clinical Programme, SingHealth Duke-NUS Academic Medical Centre, Singapore, Singapore; 3 Division of Medicine, KK Women’s and Children’s Hospital, Singapore, Singapore; 4 Children’s Intensive Care Unit, Department of Pediatric Subspecialties, KK Women’s and Children’s Hospital, Singapore, Singapore; 5 Duke-NUS Medical School, Singapore, Singapore; 6 Children’s Blood and Cancer Centre, KK Women’s and Children’s Hospital, Singapore, Singapore; 7 Yong Loo Lin School of Medicine, National University of Singapore, Singapore, Singapore; 8 Lee Kong Chian School of Medicine, Singapore, Singapore; 9 Department of Child Development, KK Women’s and Children’s Hospital, Singapore, Singapore

**Keywords:** autism spectrum disorder, children, cord blood, immune profiling, mass cytometry

## Abstract

**Introduction:**

Autism spectrum disorder (ASD or autism) is a neurodevelopmental condition characterized by social difficulties and restricted, repetitive behaviors, and is a leading cause of morbidity in children and adolescents. Immune dysregulation, including neuroinflammation, brain autoantibodies, and altered adaptive responses, has been implicated in autism pathophysiology. To address these immune alterations, autologous cord blood transfusion has been proposed as a therapy, given its abundance of immune regulatory cells and lack of rejection risk.

**Methods:**

Using multi-parametric mass cytometry, we profiled the peripheral blood mononuclear cells from children with autism at pre-transfusion and 6 months post-transfusion to assess the effects of this intervention on effector and regulatory subsets.

**Results:**

Our results showed that transfusion did not increase regulatory T or B cells. However, there was a decrease in CD4+ Central Memory (CM) T cells and memory B cells 6 months after transfusion that are not attributable to changes related to an increase in age.

**Discussion:**

Our findings suggest that autologous cord blood transfusion does not appear to provide major systemic immunoregulatory benefits in children with autism.

## 1 Introduction

Autism spectrum disorder (ASD or autism) is a heterogeneous neurodevelopmental condition, characterized by persistent difficulties in social communication and interaction, along with restricted and repetitive behaviours (RRBs) (1). Genetics, environmental exposures, and biochemical factors contribute to its etiology. Notably, there is increasing evidence that immune dysregulation plays a role in autism by affecting cerebral function and neuroinflammation (2, 3). Exposure of the developing fetal neurons to inflammatory cytokines during maternal immune activation is a risk factor for autism and other neurodevelopmental disorders, whether from autoimmune reactions or in response to bacterial or viral infections during pregnancy (4–7). Notably, autoantibodies directed against fetal brain antigens have been found in the blood of mothers of autistic children or the children themselves (8–10). Additionally, autism often coexists with other immune-mediated disorders such as type 1 diabetes, inflammatory bowel disease, allergies, and asthma (11–13). Specifically, mothers with pre-existing rheumatoid arthritis, systemic lupus erythematosus, and type 1 diabetes are also more likely to have children with autism (14–16). Collectively, this suggests an immune contribution to autism pathogenesis.

Perturbation in adaptive immune responses is observed in children with autism. Particularly, these patients have a depressed regulatory immune response and an upregulation of pro-inflammatory cytokines that correlates with disease severity (17–22). Specifically, altered T cell responses with a lower helper to suppressor T cell ratio (23–25), an increased skewing towards a T helper 2 (TH2) profile compared to TH1 (26), increased TH17 pro-inflammatory response (24, 25), and decreased regulatory T cells (Tregs) (27–29) have been reported in children with autism. Importantly, the dysregulation in inflammatory T cell responses is correlated with a poorer score in psychometric measurements of autism (20, 25). Interestingly, in autism, changes in the B-cell compartment are also found with an increased mature or activated B-cell population (27) and decreased regulatory B cells (Bregs) (28), the latter of which is associated with more severe autism.

Due to the heterogeneity in autism etiology, there are currently no curative, targeted therapies for autism. Rather, treatments focus on minimizing the autism symptoms with behavioral and communication therapies. This improves functioning but does not address its underlying etiology and core symptoms. Given the current evidence for an immunopathogenic basis for autism, immunomodulation has been proposed as a plausible therapeutic option. This includes the transfusion of umbilical cord blood (UCB), which contains stem cells with high regenerative potential (CD34^+^ and CD105^+^), as well as a significant population of cells co-expressing CD4 and CD25 (Tregs), suggesting an ability to modulate the compromised regulatory immune system in patients with autism (30–33). UCB transfusion may augment immune dysfunction through suppressing T and B cell pro-inflammatory activity and stimulating the proliferation of hippocampal neural stem cells (34, 35). Importantly, as autologous UCB (AUCB) is derived from own self, there is no risk of graft rejection or graft versus host disease.

Clinically, the transfusion of allogenic cord blood mononuclear cells (CBMNCs) and umbilical cord-derived mesenchymal stem cells (UCMSCs) in children with autism was found to be effective in improving symptoms of autism short-term for up to 16 weeks post-treatment (36). Similarly, a phase I open-label study on a single intravenous (IV) AUCB infusion in children with autism reported statistically significant improvement in autism symptoms after 6 and 12 months (37). However, more recent double-blinded randomized controlled trials on a single IV AUCB (38) or both autologous and allogenic UCB (39) for children with autism showed that although UCB infusion was safe, no significant improvement in psychometric outcome measures was found after six months. Similarly, an open-label within-subjects study on a single dose of AUCB transfusion in autistic children showed that although there were statistically significant improvements in autism severity scores in the first 6 months post-transfusion, these findings were not sustained over the long term, and inconsistencies between the different psychometric measures were found (40). Overall, these studies show that although UCB transfusion is relatively safe, its clinical benefit for autism is unclear.

Immunologically, AUCB transfusion in children with type 1 diabetes significantly increased overall Tregs and naive Tregs 6 and 9 months after transfusion, respectively (41). AUCB transfusion in patients with chronic GVHD has also been reported to promote robust recovery of interleukin-10 (IL-10)- producing Bregs (B10 Bregs), which promote suppressive activity against CD4^+^ T cells with alleviation of GVHD complications (42).

Henceforth, we aimed to study the peripheral blood mononuclear cells (PBMCs) from children with autism with multiparametric mass cytometry, as an immune monitoring platform, to depict the post-AUCB transfusion immunome at 6-months post-infusion. Our null hypothesis was that there is no increase in the immunoregulatory cells, namely Tregs and Bregs, after AUCB transfusion. Unexpectedly, we identified a decrease in CD4^+^ CM T cells and memory B cells 6 months after receiving the AUCB transfusion. However, contrary to previous studies, we found no significant changes in the regulatory immune cells in our study.

## 2 Materials and methods

### 2.1 Sample origin and cell isolation

Peripheral blood was obtained from children with autism (N = 19) recruited through a clinical trial on autologous umbilical cord blood infusion for the treatment of autism in young children (registered with the Singapore Health Sciences Authority Clinical Trials Register with Certificate No. CTC1800071), now published (40). Children who met all of the following clinical criteria were recruited: (A) age ≥24 months to ≤60 months, and ≤72 months at time of planned AUCB infusion (6 months after the first assessment), (B) confirmed diagnosis of autism based on the DSM-5 diagnostic criteria, using the Autism Diagnostic Observation Schedule, Second Edition (ADOS-2), and an autism-specific interview or Autism Diagnostic Interview, Revised (ADI-R), (C) AUCB available from a cord blood bank, with a minimum pre-cryopreservation total nucleated cell count (TNCC) of 25 million cells/kg of subject body weight, (D) stable on regular early intervention and any medications for at least 6 months before AUCB transfusion, and (E) normal results for chromosomal karyotyping and Fragile X genetic screening. The exclusion criteria were: (A) history of prior cell therapy, (B) use of IV immunoglobulins or other anti-inflammatory medications, (C) genetic/metabolic abnormalities or dysmorphic features, (D) history of unstable epilepsy, (E) sensory or motor impairment, (F) uncontrolled infection, or (G) active malignancy. Peripheral blood was collected before AUCB transfusion (T0) and 6 months after AUCB transfusion (T6), and peripheral blood mononuclear cells (PBMCs) were isolated using Ficoll-Paque PLUS (GE Healthcare, UK) density centrifugation as per the manufacturer’s instructions. PBMCs were subsequently cryopreserved in human serum (HS, Gibco, USA) with 10% (v/v) dimethyl sulfoxide (DMSO, Sigma-Aldrich, UK).

Autism diagnoses were conducted by ADOS- and ADI-R-trained clinical psychologists and/or developmental behavioral pediatricians and typically included detailed parent/caregiver interviews as well as teacher feedback via structured forms for children attending a preschool or school. Translated versions were not required as the caregivers were all fluent in English. Children had to meet the cut offs for an autism spectrum diagnosis on the ADOS and ADI-R (if used) and display directly observed symptoms meeting the DSM-5 diagnostic criteria for ASD.

Fevers, infections, and major medical events during the 6-month follow up after AUCB transfusion were recorded and it was noted that infections were rare and self-limiting, typical of common childhood infections. The vaccination records of the subjects were not available.

Age-matched healthy control (HC) samples consisted of peripheral blood collected during intravenous cannulation pre-operatively from otherwise healthy children without inflammatory diseases (active infections and rheumatological diseases) undergoing elective surgeries. Healthy PBMCs were examined with CyTOF (N = 23).

### 2.2 Mass cytometry

Frozen PBMCs were thawed in Roswell Park Memorial Institute 1640 (RPMI) medium supplemented with 10% (v/v) Human Serum (Corning, USA), 1x (v/v) Penicillin-Streptomycin-Glutamine (Gibco, USA), and Benzonase (1:1000). Cells were then resuspended in RPMI with 10% (v/v) Human Serum and 1x (v/v) Penicillin-Streptomycin-Glutamine and rested for 30 minutes at 37°C. After rest, the cells were harvested, and samples were divided equally into tubes for two separate stimulation conditions, for staining using Panel A and Panel B (**Supplementary Tables 1**, **2**). Panel A was designed for the evaluation of the entire immunome, while Panel B characterizes the B cell compartment with greater detail.

A minimum of 0.75 million cells per sample was used for each stimulation condition; for insufficient cell counts, only stimulation and staining for Panel B were conducted. For Panel A stimulation, blocking was first done with anti-CD40 blocking antibody (0.5 µg/ml) for 15 minutes at 37°C. Following that, stimulation was done with phorbol 12-myristate 13 acetate (PMA) at 150 ng/ml and ionomycin at 250 ng/ml (Sigma-Aldrich, UK) together with staining with CD107a-PE antibody (1:50 dilution) for 5 hours. Brefeldin A and Monensin (1:1000 dilution) (eBioscience) were added during the last 3 hours of the incubation for blockade of protein transport. For Panel B stimulation, cells were primed by incubation with CpG DNA (1 µg/ml), anti-hemagglutinin (HA) (5 µg/ml), and HA-tagged CD40L (1 µg/ml) for 24 hours. Stimulation using Brefeldin A (1:1000 dilution), PMA (50 ng/ml), and ionomycin (1 µg/ml) was done for the last 5 hours of the incubation. Due to insufficient cell numbers, differing sample counts were used for Panel A (T0: N = 17, T6: N = 17) and Panel B (T0: N = 18, T6: N = 19).

After stimulation, cells were washed once with cell staining buffer (CSB) (Phosphate Buffered Saline (PBS) with 4% HI-FBS, 2 mM EDTA, 0.05% sodium azide) and centrifugated at 600g for 3 minutes at 4°C. Samples were decanted and stained with cisplatin viability stain (PBS with 10 μM cisplatin) (DVS Sciences, USA) for 5 minutes on ice. Cells were washed and stained with CD45 antibody conjugated with lanthanide metal-89, 106, 113 or 115 – a quadruplet barcode system (used at 10 μg/ml each) in a 100 μl final reaction volume for 20 minutes on ice. Staining using 5 μl fluorescein isothiocynate (FITC) anti-human TCR γ/δ (Invitrogen, USA) was done on Panel A samples only. Afterwards, cells were washed three times and combined before staining with the two corresponding antibody panels, Panel A and Panel B (**Supplementary Tables 1**, **2**).

Surface marker antibodies conjugated with lanthanide metal with a final reaction volume of 150 μl were added accordingly to each tube and stained at room temperature for 15 minutes. Following surface staining, cells were washed once with CSB and then once with 1x PBS to remove any residual unbound antibodies prior to fixation and permeabilization. The cells were resuspended in 1 ml of fixation/permeabilization mix buffer (eBioscience, USA) prepared following the manufacturer’s instructions and incubated for 45 minutes on ice. Subsequently, cells were washed twice with permeabilization wash buffer (eBioscience, USA) prepared according to the manufacturer’s instructions and centrifugated at 840g for 6 minutes before decanting the wash buffer. All centrifugation steps after this were at 840g for 6 minutes at 4°C. Intracellular marker antibodies conjugated with lanthanide metal with a final reaction volume of 150 μl were added accordingly to each tube and stained on ice for 40 minutes. After staining, the cells were washed once with permeabilization wash buffer and then resuspended in 1x PBS with 1.6% Paraformaldehyde (PFA) for up to 2 days at 4°C until acquisition.

On the day of data acquisition, the cells were pelleted and stained with 500 μL of DNA intercalator (DVS Sciences, USA) diluted in 1.6% PFA in 1x PBS at room temperature for 20 minutes. The cells were then washed once with CSB and resuspended in Maxpar Cell Acquisition Solution (CAS) Plus for CyTOF XT (CAS+) (Standard BioTools, USA) for counting. Cells were then resuspended to a density of 0.65 million per mL in CAS+ and pelleted. Samples were acquired on CyTOF XT with 10% (v/v) EQTM Four Element Calibration Beads (43) (Standard BioTools, USA) used for calibration of each sample.

### 2.3 Processing and analysis of data output from CyTOF XT mass cytometer

CyTOF FCS data collection, conversion, randomization, beads normalization, and concatenation were performed through the manufacturer’s software (CyTOF Software 6.7, Fluidigm). The live single cell events (singlets) were gated based on a plot of DNA intercalator versus event length, and live cell events negative for cisplatin were obtained as previously described (44). De-barcoding was done using bivariate gating strategy in FlowJo 10.7.1 (Becton, Dickinson & Company, USA) and exported for unsupervised and supervised analysis.

### 2.4 Quality control measures

Due to the large number of samples, the samples were stained and interrogated with the mass cytometer in different batches. To minimize batch effect, quality control measures were incorporated into the study. A sufficient amount of metal-conjugated antibodies was prepared for all runs to prevent variation from different antibody batches. Each run included both time points, ensuring that any variation will affect all time points to the same degree. One aliquot of PBMCs from the same healthy donor was used for each CyTOF run as an internal biological control to identify batches with significant differences in marker expression profiles. Batch-wise standard scaling was conducted before clustering and downstream analysis to exclude outliers and reduce variance.

### 2.5 Immune maps

Immune maps were created and analyzed using the web-based data mining tool sciAtlasMiner (single-cell immune Atlas Miner) within the online EPIC Immune Atlas Interactive Web Portal (45). CyTOF data in FCS format were first merged into a single CSV file. Sample parameters (i.e. age, gender, and time point) were encoded in the file names and extracted by pattern matching. Batchwise scaling was conducted as a pre-processing step to mitigate the effect of batch variations between the different CyTOF runs. To identify cell populations in an unsupervised manner, datasets were first subjected to hyperbolic arcsine transformation with a scale factor of 5 (*asinh5*). Subsequently, flow cytometry analysis using self-organized maps (FlowSOM) (46) clustering was applied using an 8x8 grid size. Batchwise standard scaling was applied after *asinh5* to assign equal weights to all features and reduce technical variations.

Assisted multi-layer cluster annotation and generation of dendrogram heat maps were conducted. Cell types and relationships were first automatically assigned to clusters in a multi-layered hierarchical fashion, and the expression pattern of each marker was then manually verified and translated into cell types at multiple levels of detail. Up to six layers of detail were added, and closely related clusters were merged. The cell-type annotations were transferred to the immune maps for downstream data mining. We performed non-linear dimensionality reduction using t-distributed stochastic neighbor embedding (tSNE) to visualize multi-dimensional marker expression landscapes in two dimensions. The expression pattern classifications were verified manually and translated into cell types at multiple levels of detail, allowing lassoing of individual cell types in the tSNE plot.

Expression heat map was obtained by exporting *asinh5*-transformed median marker expression of the most detailed phenotypic layer and manually scaling marker expression relative to lowest- and highest-expressing cell types (value from 0 to 1). The normalization steps took into account cell subsets that were not shown in the final heatmap (e.g. B cells), thus providing a thorough picture of global marker expression.

### 2.6 Statistical analysis

The false discovery rate (FDR) was set at q<0.1 due to the exploratory nature of the study. For comparisons within the autism cohort across time points (T0 and T6), the parametric, paired, two-tailed t-test was performed. For the comparison between HC and children with autism before AUCB transfusion (T0), the parametric, unpaired, two-tailed t-test was performed. For correlation analysis between age and cell type abundance, calculation of the Pearson correlation coefficient was conducted.

### 2.7 Study approval

Ethical approval from the SingHealth Centralised Institutional Review Board was obtained for this study (CIRB ref. no. 2017–2620 and 2019-2961). Samples from both groups were obtained with written informed consent prior to participation.

### 2.8 Data availability

Relevant data are available upon reasonable request.

## 3 Results

### 3.1 The post-AUCB infusion immunome revealed a decrease in CD4^+^ central memory T cells

To characterize the immunome, we studied the circulatory immune cell composition of children with autism before AUCB transfusion (T0), and 6-months after transfusion (T6). This single-center cohort consists of 19 children who received AUCB infusion as a treatment for autism (40). In the published study, it was reported that at T6 (T = 12 months in the published paper), there were no statistically significant improvements in the Vineland Adaptive Behavior Scale, Third Edition (Vineland-3), Expressive One-Word Picture Vocabulary Test, Fourth Edition (EOWPVT-4), Stanford Binet Intelligence Scale, Fifth Edition (SB-5), Brief Observation of Social Communication Change (BOSCC), Sensory Experience Questionnaire, Version 2.1 (SEQ-2.1) and Clinical Global Impression-Severity (CGI-S) scores. Although there were some statistically significant improvements in selected subscale scores of the Child Behavior Checklist (CBCL), Pervasive Developmental Disorder-Behavior Inventory (PDDBI), and the Repetitive Behavior Scale-Revised (RBS-R), these were not in consistent in direction across the different measures and not sustained over a longer follow-up period except for Clinical Global Impression-Improvement (CGI-I).

**Figure 1:**
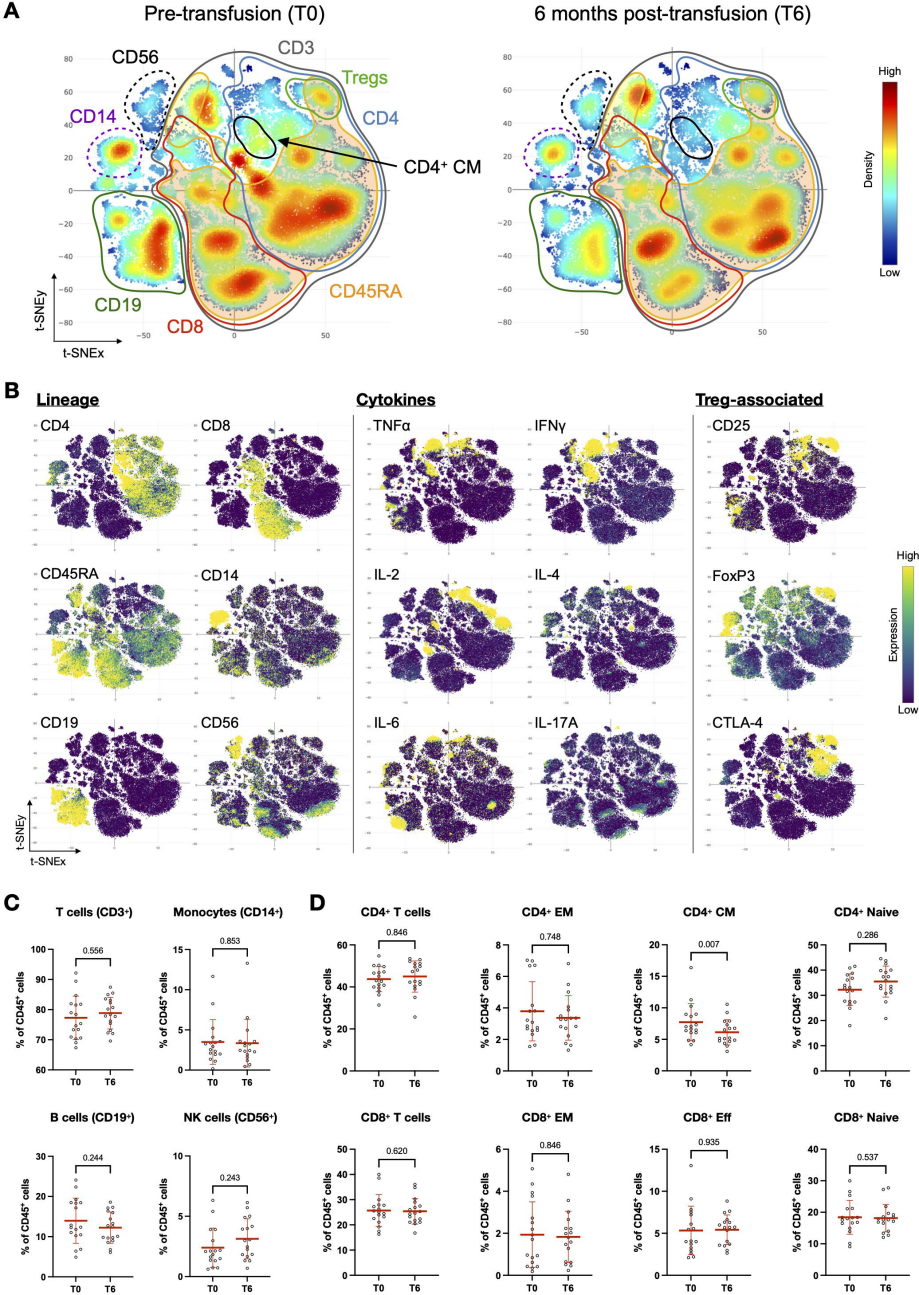
The post-AUCB infusion immunome revealed a decrease in CD4^+^ central memory T cells. **(A)** Density plots consisting of a random sampling of 50, 000 events per time point from PBMCs from children with autism pre- and post-AUCB transfusion analyzed with t-SNE. Dimensional reduction with t-SNE of the global immunome of children with autism pre-AUCB transfusion (T0, N = 17) and 6-months post-transfusion (T6, N = 17) after AUCB transfusion. **(B)** Density plots for marker expression in t-SNE map. Density expression maps showing the distribution and expression of cells with the indicated markers. **(C)** Mean frequency changes of major immune cell lineages from T0 to T6, expressed as a percentage of total CD45^+^ cells. Data is presented as mean ± SD. Statistical significance was calculated using paired t-test. NK, natural killer. **(D)** Mean frequency changes of key T cell subsets from T0 to T6, expressed as a percentage of total CD45^+^ cells. Data is presented as mean ± SD. Statistical significance was calculated using paired t-test. EM, effector memory, CD45RA^-^CCR7^-^; Eff, effector, CD45RA^+^CCR7^-^; Naive, CD45RA^+^CCR7^+^; CM, central memory, CD45RA^-^CCR7^+^; Tregs, Regulatory T cells, CD4^+^CD25^+^FoxP3^+^. For this data, Panel A was used for PBMCs staining.

The clinical characteristics and psychometric outcome scores of these children with autism are shown in **Table 1**. We interrogated the PBMCs with 2 mass cytometry panels (A and B) consisting of 43 markers each, and a CD45 barcoding strategy to pool 14 samples into a single experimental setup to reduce technical variation (44) (**Supplementary Tables 1**, **2**). Each sample is randomly down-sampled to 10, 000 live, single-cell events for clustering and dimensional reduction with t-SNE for cell subsetting and data visualization, respectively (refer to the Methods section for more details). The t-SNE plots illustrate the immunome at pre-transfusion (T0) and 6 months post-transfusion (**Figures 1A, B**).

**Table 1:** Study cohort. Baseline characteristics of study participants.

Child characteristics	N = 19
Age at baseline in years	
Mean (SD)	4.15 (0.62)
Gender, *n* (%)	
Male	15 (78.9)
Female	4 (21.1)
Ethnicity, *n* (%)	
Chinese	12 (63.2)
Malay	0 (0.0)
Indian	4 (21.0)
Others	3 (15.8)
Weight at time of infusion in kg	
Mean (SD)	18.76 (2.38)
ADOS-2 module used, *n* (%).	
Module 1	16 (84.2)
Module 2	3 (15.8)
Module 3	0 (0.0)
IQ standard scores, mean (SD)	
Non-verbal IQ	67.5 (17.7)
Verbal IQ	59.2 (15.0)
Full scale IQ	62.7 (17.4)
Psychometric outcome measures	
Vineland-3 standard scores	
Adaptive Behaviour Composite	
Baseline, mean (SD)	62.4 (9.9)
Mean difference at T6, estimate (95% CI)	-1.23 (-4.99, 2.53)
Socialization domain	
Baseline, mean (SD)	57.4 (7.7)
Mean difference at T6, estimate (95% CI)	2.08 (-5.82, 9.98)
PDDBI Total T-score	
Baseline, mean (SD)	52.5 (10.1)
Mean difference at T6, estimate (95% CI)	7.11 (-10.70, 24.92)
EOWPVT-4 raw score	
No. non-verbal at baseline, *n* (%)	7 (36.8)
Baseline, mean (SD)	19.6 (21.9)
Mean difference at T6, estimate (95% CI)	-3.42 (-11.31, 4.48)
CGI Overall Severity score	
Baseline, mean (SD)	5.3 (0.73)
Mean difference at T6, estimate (95% CI)	-0.08 (-0.91, 0.76)

Vineland-3, Vineland Adaptive Behavior Scale, Third Edition; PDDBI, Pervasive Developmental Disorder-Behavior Inventory; EOWPVT-4, Expressive One-Word Picture Vocabulary Test, Fourth Edition; CGI, Clinical Global Impression; SD, standard deviation; 95% CI, 95% confidence interval; T6, 6-months post-transfusion.

From the 2-dimensional t-SNE density plots, there were subtle changes in the immunome of patients with autism post-AUCB infusion (**Figure 1A**). To depict the immune landscape, the PBMCs were stimulated with phorbol 12-myristate 13 acetate (PMA) and Ionomycin for 5 hours before staining with the mass cytometry panel A. We assessed the frequencies of the major immune lineages as a proportion of total CD45^+^ cells. There were no significant changes in the CD3^+^ T cell, CD14^+^ monocyte, CD19^+^ B cell, and CD56^+^ Natural Killer (NK) cell populations (**Figure 1C**). Although the total CD4^+^ T cell frequency is unchanged at T6, we observed a significant decrease in the CD4^+^ central memory (CM) subset, defined as CD45RA^-^CCR7^+^, at T6 (p=0.007) (**Figure 1D**). No significant changes were found in the effector memory (EM, CD45RA^-^CCR7^-^) and naive (CD45RA^+^CCR7^+^) CD4^+^ T cells, as well as the EM, effector (Eff, CD45RA^+^CCR7^-^), and naive CD8^+^ T cells (**Figure 1D**). To determine if this increase is due to age-related immune maturation over 6 months (from T0 to T6), we re-mined an existing published mass cytometry dataset of healthy children of comparable ages to our current cohort with supervised analysis using bivariate gating (N = 53; mean age: 5.93 years old; SD: 1.73) (45). Our analysis showed that CD4^+^ CM cells increased with age, both as a proportion of total CD45^+^ cells (r=0.394, p=0.004) and as a proportion of CD4^+^ cells (r=0.373, p=0.006) (**Supplementary Figure 1A**). Hence, the decrease in CD4^+^ CM T cell population observed 6 months after AUCB transfusion is not attributable to a natural age-related reduction within this age range.

### 3.2 The post-AUCB infusion decrease in CD4^+^ CM cells was due to a nominal reduction in the CCR6^-^CXCR3^-^CXCR5^-^ CD4^+^ CM T cell frequency

**Figure 2:**
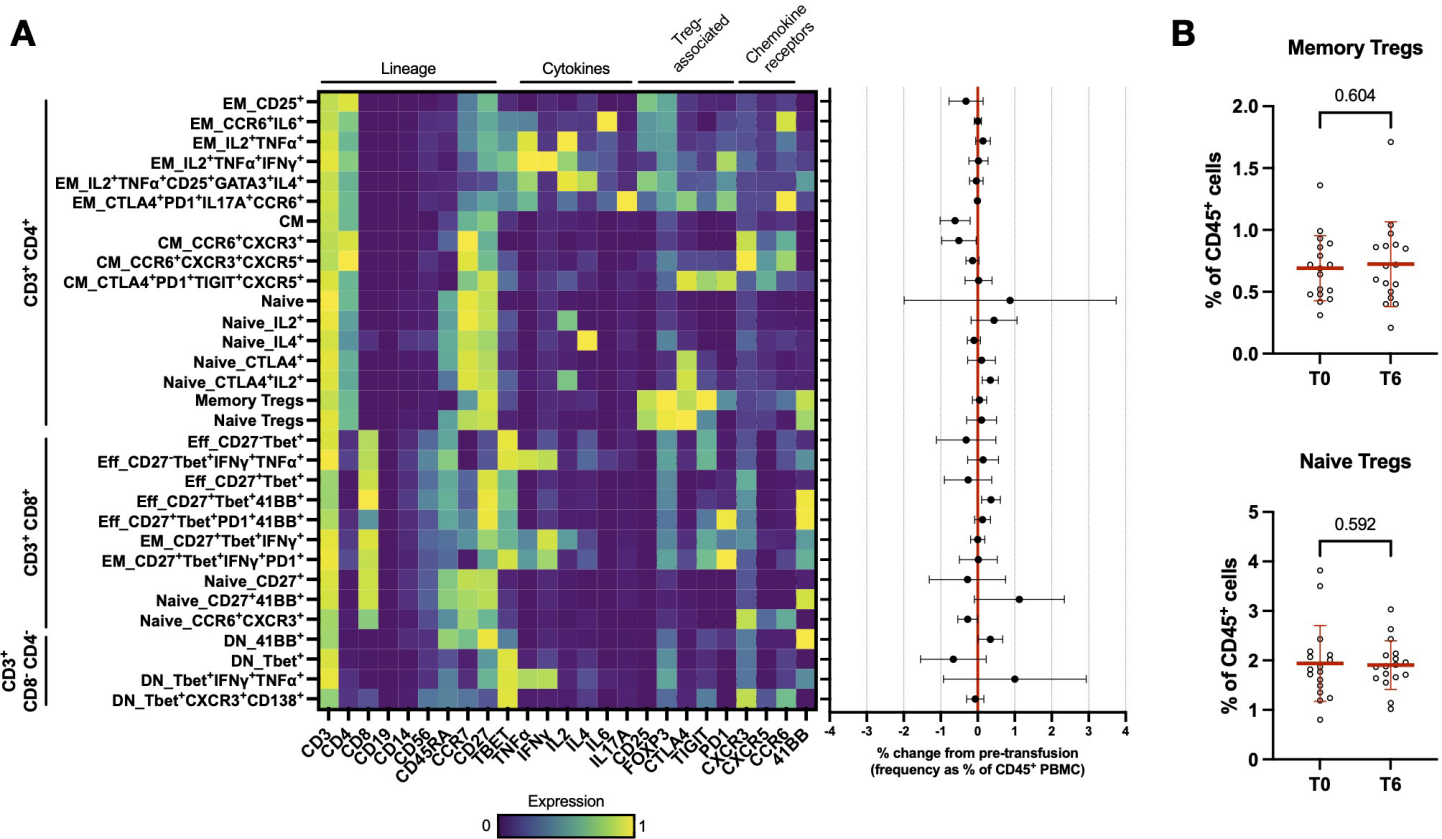
Representative studies were chosen based on the use of flow cytometry. **(A)** Expression heat map and mean frequency changes of 31 phenotypically distinct T cell subsets. Marker expression was normalized on a scale from 0 (lowest) to 1 (highest). Percentage changes for each subset are reported and expressed as the mean difference compared to T0 (pre-transfusion), and 95% confidence interval of the mean difference. Statistical significance was calculated using paired t-test with 10% FDR. No tests met significance at FDR q<0.1. **(B)** Mean frequency changes of Tregs (CD4^+^CD25^+^FoxP3^+^) from T0 to T6, expressed as a percentage of total CD45^+^ cells. Memory Tregs were defined as CD45RA^-^CCR7^-^ and naïve Tregs were defined as CD45RA^+^CCR7^+^. Data is presented as mean ± SD. Statistical significance was calculated using paired t-test. For this data, Panel **(A)** was used for PBMCs staining, for pre-AUCB transfusion (T0, N = 17) and 6-months post-transfusion (T6, N = 17).

As mass cytometry enables deep immunophenotyping, we examined in greater detail the changes within the T cell compartment. An unbiased clustering approach with FlowSOM followed by merging of closely related nodes reveals 31 distinct and unique T cell subsets (**Figure 2A**). Due to the exploratory nature of the study, we used a false discovery rate (FDR) cut-off of <10% (q<0.1) and found that none of these 31 subsets were significant. We were able to segregate the CD4^+^ CM cell population into 4 distinct subsets based on the differential expression of 3 chemokine receptors, CCR6, CXCR3 and CXCR5. The CD4^+^ CM T cell subset that showed the greatest reduction from pre-transfusion (T0) was CCR6^-^CXCR3^-^CXCR5^-^. Two subsets, a CCR6^-^CXCR3^-^CXCR5^-^ CD4^+^ CM T cell (annotated on **Figure 2A** as CM) and CTLA4^+^IL2^+^ naive CD4^+^ T cell subsets, had a q-value of exactly 0.1. Beyond this, there were no significant changes detected in the CD4^+^ T helper cell subsets involved in TH1, TH2, and TH17 responses (characterized by the production of IFNɣ, IL-4, and IL-17A, respectively), as well as T follicular helper cells (defined as CXCR5^+^) and Treg cells (CD25^+^FoxP3^+^). Of note, there were no observable changes in the memory Tregs (CD45RA^-^CCR7^-^; mean difference: 0.0473; 95% CI: -0.144, 0.239; p=0.604) or naive Tregs (CD45RA^+^CCR7^+^; mean difference: 0.103; 95% CI: -0.301, 0.507, p=0.592). The change in memory Tregs as a percentage of total CD45^+^ cells from T0 to T6 was 0.691% (SD: 0.263) to 0.724% (SD: 0.343), and the change in naive Tregs was 1.940% (SD: 0.767) to 1.908% (SD: 0.492) (**Figure 2B**). In summary, we observed that children with autism treated with AUCB transfusion had a general decrease in CD4^+^ CM at T6 (**Figure 1D**), attributable to a nominal reduction in the CCR6^-^CXCR3^-^CXCR5^-^ CD4^+^ CM T cell subset (**Figure 2A**).

### 3.3 B cell changes post-AUCB transfusion

**Figure 3:**
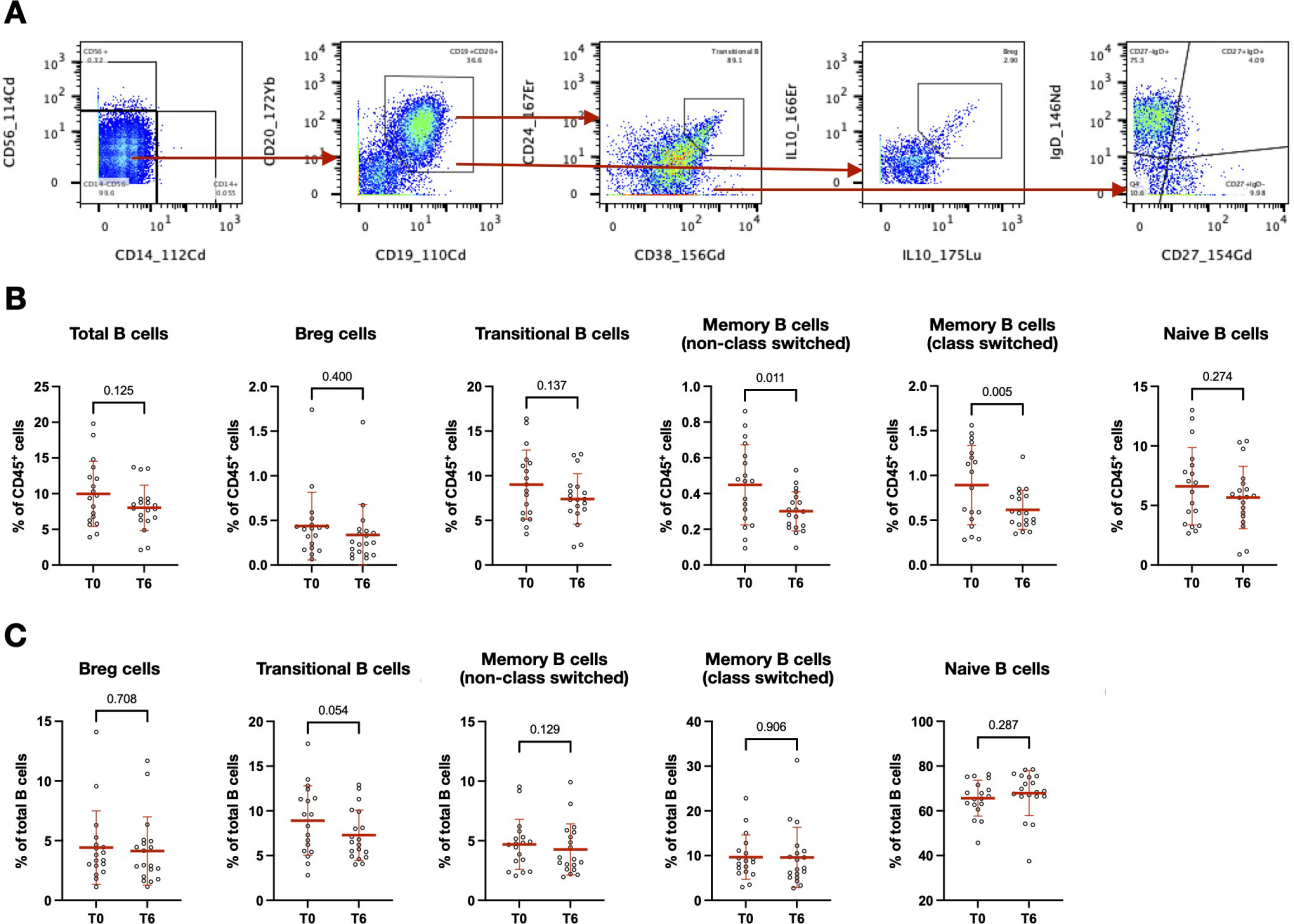
B cell changes post-AUCB transfusion. **(A)** Supervised bivariate gating strategy for B cell subsets. The gating strategy is shown for the relevant cell subsets described in accordance with the following markers: CD14, CD56, CD3, CD19, CD20, CD24, CD38, CD27, IgD, and two different IL-10 stains. **(B)** Mean frequency changes of B cells and B cell subsets as a percentage of total CD45^+^ cells from T0 (N = 18) to T6 (N = 19). Data is presented as mean ± SD. Statistical significance was calculated using paired t-test. **(C)** Mean frequency changes of B cell subsets as a percentage of total B cells (CD19^+^) from T0 to T6. Data is presented as mean ± SD. Statistical significance was calculated using paired t-test. Bregs: regulatory B cells, double-positive for IL-10; transitional B cells: CD24^+^CD38^+^; memory B cells (non-class switched): CD27^+^IgD^+^; memory B cells (class switched): CD27^+^IgD^-^; naïve B cells: CD27^-^IgD^+^. For this data, Panel B was used for PBMCs staining.

Next, we examined the B cell compartment with supervised bivariate gating on the dataset derived with mass cytometry panel B (**Figure 3A**). Panel B contained a greater number of B-cell-specific markers to comprehensively characterize the B cells (**Supplementary Table 2**). Additionally, the PBMCs were stimulated with a specific cocktail for 24 hours to enable the identification of Breg cells, defined as IL-10 double-positive, unequivocally using two different clones of anti-human IL-10 antibodies (**Figure 3A**). With this protocol, we did not observe any significant changes in total B cells, as well as Breg (IL-10 double-positive), transitional (CD24^+^CD38^+^), and naive (CD27^-^IgD^+^) B cell levels between T0 and T6 (**Figure 3B**). However, there was a significant decrease in both non-class switched (CD27^+^IgD^+^) and class switched (CD27^+^IgD^-^) memory B cells at T6 (**Figure 3B**). Next, we examined the B cell composition by evaluating the different B cell subsets as a proportion of total B cells (CD19^+^). There were no significant changes in any of the B cell subsets, suggesting that the composition of the B cell compartment was unchanged after AUCB transfusion (**Figure 3C**).

Again, this decrease in non-class switched and class switched memory B cells did not appear to be age-related (non-class switched memory B cells: r=0.207, p=0.137; class switched memory B cells: r=0.071, p=0.615) (**Supplementary Figure 1B**). In summary, we observed a reduction of both non-class switched and class switched memory B cells from T0 to T6 that may be attributed to AUCB transfusion.

### 3.4 Differences in the immunome of healthy children and children with autism

**Figure 4:**
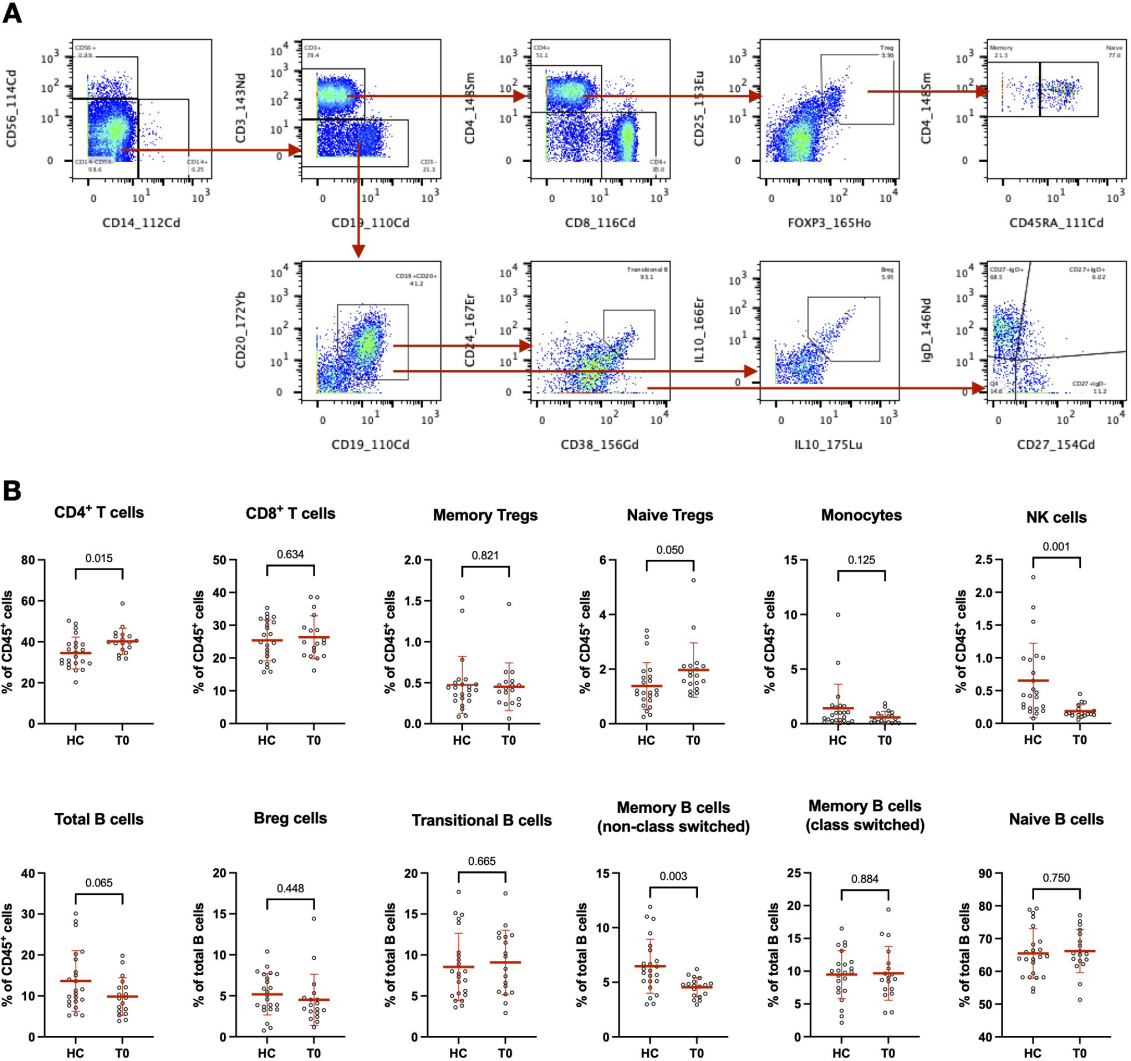
Differences in the immunome of healthy children and children with autism. **(A)** Supervised bivariate gating strategy for cell subsets. The gating strategy is shown for the relevant cellular subsets described in accordance with the following markers: CD14, CD56, CD3, CD4, CD8, CD45RA, CCR7, CD25, FoxP3, CD19, CD20, CD24, CD38, CD27, IgD, and two different IL-10 stains. **(B)** Mean frequency differences of cell subsets between healthy controls (HC, N = 23) and children with autism before AUCB transfusion (T0, N = 18). Data from CD4^+^ T cells, CD8^+^ T cells, memory Tregs, naive Tregs, monocytes, NK cells, and B cells is expressed as a percentage of total CD45^+^ cells. Data from Breg cells, transitional B cells, memory B cells (non-class switched and class switched), and naive B cells is expressed as a percentage of total B cells (CD19^+^). Data is presented as mean ± SD. Statistical significance was calculated using paired t-test. For this data, Panel B was used for PBMCs staining.

To delineate the differences in the immune cell composition between children with autism before AUCB transfusion and in health, we utilized the mass cytometry data from Panel B due to the availability of a larger dataset from both healthy controls (HC, N = 23) and children with autism at T0 before AUCB transfusion (N = 18) for analysis. The populations were examined with supervised bivariate gating (**Figure 4A**).

The plots of the immune cell populations are shown in **Figure 4B**. In the T cell compartment, there was a significant increase in CD4^+^ T cells (p=0.015) in children with autism compared to healthy controls, but no significant differences were found for CD8^+^ T cells, memory Tregs, and naive Tregs. In the B cell compartment, there was a significant decrease in non-class switched memory B cells (p=0.003) as a proportion of total B cells in children with autism, but no changes in Breg cells, transitional B cells, class switched memory B cells, naive B cells, or total B cells. Finally, children with autism had significantly lower NK cells (p=0.001), but no differences in monocytes.

## 4 Discussion

In this study, we utilized multi-parametric mass cytometry as an immune-monitoring platform to study the immunome of children with autism after an AUCB transfusion used as a therapeutic intervention by examining both the effector and regulatory arms of the immune system. We identified a general decrease in CD4^+^ CM T cells 6 months post-AUCB transfusion in these children. This decrease did not translate to a significant change in the total CD4^+^ T cell population (expressed as a frequency of total CD45^+^ cells). This is likely due to the relatively smaller contribution of CD4^+^ CM cells as a proportion of total CD4^+^ cells. We determined that the decrease in CD4^+^ CM cells was not due to a natural developmental phenomenon; a concern, as there was a six-month lapse from the T0 immune profile to that at T6. This was supported by existing published normograms of immune cell frequencies across the relevant age group of our study showing that the proportion of CD4^+^ CM cells increased over the age of 4 to 18 years (47, 48) and our analysis of an existing mass cytometry dataset acquired from healthy children (45) that also showed an increase in the CD4^+^ CM T cells with age. Although the 6-month time frame in our study is much shorter than those published, these reference values indicate that CD4^+^ CM levels tend to increase with age. Strikingly, our observed change in CD4^+^ was in the inverse direction of the CD4^+^ CM trends in healthy populations, suggesting that the observed effect at T6 may be due to the AUCB transfusion itself.

Nevertheless, as this clinical trial was conducted over the children’s school-going age as well as over the duration of the COVID-19 pandemic, the possibility that the immune changes observed are due to environmental factors cannot be fully excluded. While the gender allocation of our study has matched the incidence of autism in the general population, the clinical study was underpowered to detect statistically significant differences based on gender (40). However, variations in lymphocyte subsets and immune responses have been shown in children (49–51), and thus the effects of AUCB transfusion may manifest differently for male and female children.

We evaluated the possibility that the pre-medications administered during AUCB transfusion, namely diphenhydramine, hydrocortisone, ondansetron, and oral chloral hydrate (40), could have contributed to the decrease in CD4^+^ CM T cells. Hydrocortisone administration was shown to reduce CD4^+^ CM T cell levels in the peripheral blood due to upregulation of CXCR4 expression, leading to recruitment and accumulation in the bone marrow (52–54). However, this is a pan-T cell effect and is unlikely to affect only CD4^+^ CM T cells in isolation. On the other hand, ondansetron was shown not to exert an effect on CXCR4/CXCL12-mediated migration (55). There were no reported effects of diphenhydramine or chloral hydrate on CD4^+^ CM T cells. Nonetheless, any effects from the pre-medications are unlikely to last up to 6 months post-transfusion. Taken together, it is unlikely that the changes observed were due to the pre-medications used before AUCB transfusion.

As cord blood is enriched with naive T cells (56), a decrease in CM T cells suggests a delayed maturation of naive CD4^+^ T cells to the memory phenotype. However, studies show that the shift from naive to CM phenotype occurs rapidly within 2 to 5 months of CB transplantation (57–59). Nevertheless, while not a CD4^+^ CM-specific characteristic, T cell repertoires were also found to return to normal only 12 to 18 months after UCB transplantation (60), and delays in T cell recovery were also found to be delayed after UCB transfusion compared to bone marrow transplantation (61) or peripheral blood stem cell transplantation (62) suggesting a possible delay in the development of naive CD4^+^ T cells in the infused UCB. Importantly, these studies examine the effect of allogenic UCB transplantation, and thus far, there have not been studies reporting changes in CD4^+^ CM T cells after AUCB transfusion. While the reason for the decline in CD4^+^ CM T cells after AUCB transfusion is unclear, there is a possibility that the observed effect is due to a delayed development or maturation of naive CD4^+^ T cells after AUCB transfusion.

Although there were no significant changes in the total B cell level 6 months after AUCB transfusion, there was a significant decrease in memory B cells as a proportion of total PBMCs. Again, this may be due to the naive nature of the infused AUCB. However, when expressed relative to the B cell population, there were no significant changes in any of the B cell subsets analyzed. This could be explained by a slight decreasing trend of total B cells that masked the decrease in the memory B cell subsets. Although we did not observe an age-related decrease in either memory B cell subset in the published dataset we analyzed, memory B cells generally follow an increasing trend over age due to the development of the immune system as it is exposed to more antigens.

The decrease in CD4^+^ CM T cells and memory B cell subsets together point to the naive nature of AUCB (56), but together suggest a possible attenuation of immune maturation in children with autism who receive a transfusion of AUCB. Further studies of immune profiles over the longer term are required to examine the pattern of immune development and whether it is significantly affected by AUCB transfusion.

The clinical outcomes of AUCB transfusion are available (40). There were no significant improvements in autism symptoms or adaptive skills at T6, corresponding to the absence of changes in major immune lineages at the same time point. Although the clinical study reported longer-term outcomes at 12 months post-transfusion with AUCB (T12; T = 18 in the published paper), a study blood sample was not collected at that time point. However, CGI-I and EOWPVT-4 scores indicated an overall improvement in autism symptoms and language skills at T12, while Vineland-3 and PDDBI scores suggested there were no significant changes in adaptive skills and social communication. Importantly, the scores varied greatly between children, suggesting that not all children may benefit from this treatment. These inconsistencies result in difficulties detecting any contribution of immunological changes to autism pathology. Additionally, although the CGI-I Overall scores are available, they were not used as a primary outcome measure, and the small sample size of the trial prevents meaningful stratification between responders and non-responders. Therefore, we are unable to determine whether the immunological changes observed in this study correlate with changes in autism symptoms in the same cohort.

Interestingly, we did not observe any increase in Tregs or Bregs in children with autism after AUCB transfusion, contrary to what was reported in other diseases. Given that these were the main plausible therapeutic effects of AUCB transfusion on autism, and taken together with the absence of clinical benefits seen in the preceding clinical trial (40), our results do not provide evidence that AUCB infusion had any perceivable effect on the frequency of well-characterised immunoregulatory cells, Tregs and Bregs.

Several immune system abnormalities have been reported in autism, including altered T cell responses and B cell activation (reviewed in the Introduction). In our cohort, we observed a significant increase in CD4^+^ T cells in autistic children compared to healthy controls. While most studies report lower or unchanged CD4^+^ T cells in autistic populations (26, 63, 64), there have been elevated CD4^+^ T cells or CD4^+^ T cell hyperactivation in autism described in certain populations as well as in mice (23, 65, 66) (**Table 2**). Strikingly, we found a highly significant decrease in NK cells in autistic children compared to HC. This is in line with a previous study showing that children with autism have a lower proportion of NK cells compared to their healthy siblings (67); however, others report higher NK cell frequencies to possibly compensate for decreased NK cell cytotoxicity and cytokine production in children with autism (27, 68, 69) (**Table 2**).

**Table 2:** Major findings and baseline characteristics of subjects in studies evaluating T cell, B cell, and NK cell frequencies, and circulating cytokine levels in autism.

Author (year)	Patients with autism		Controls		
	No. of subjects and gender, N	Age range (years)	No. of subjects and gender, N	Age range (years)	Major related findings
Ahmad (2016)	40 (Male: 30; Female: 10)	3 - 11	32 (Male: 24; Female: 8)	3 - 11	↑ Th1-, Th2-, and Th17-related transcription factors and ↓ Treg-related transcription factors
Ashwood (2011b)	66 (Male: 59; Female: 7)	3.2 - 4.3	73 (Male: 51; Female: 22)	2.7 - 4.3	↑ proliferation in response to PHA; ↑ TNF-ɑ with PHA; ↓ IFN-γ production as recall response to tetanus toxoid
Basheer (2018)	50 (Male: 48; Female: 2)	3 - 12	30 (Male: 27; Female: 3)	3 - 12	↑ activated Th17 cells; ↑ serum IL-17 concentration
De Giacomo (2021)	26 (Male: 21; Female: 5)	4.7 - 11.9	16 (Male: 12; Female: 4)	4.2 - 15.6	↓ Bregs; ↓ Tregs; no significant changes in memory B cells and NK cells
Enstrom (2008)	52 (Male: 44; Female: 8)	2.2 - 5.6	27 (Male: 22; Female: 5)	2.3 - 4.8	↑ NK cells; ↓ NK cell-mediated cytotoxicity
Heuer (2012)	42 (Male: 37; Female: 5)	5.1 - 9	31 (Male: 24; Female: 7)	4 - 9	No changes in total T cells, total B cells, naive B cells, memory IgM^+^ B cells, and memory IgG^+^ B cells
Moaaz (2019)	44 (Male: 35; Female: 9)	5 - 9.4	45 (details not reported)	5 - 9.2	↑ Th17 cells; ↓ Tregs; ↑ Th17/Treg ratio
Mostafa (2010)	30 (Male: 22; Female: 8)	4 - 12	30 (Male: 22; Female: 8)	4 - 12	↓ Tregs (CD4^+^CD25^high^)
Nie (2022)	82 (Male: 67; Female: 15)	3.3 - 7.1	50 (Male: 44; Female: 8)	3.2 - 6.9	↑ Th1, Th2, and Th17 cells; ↓ Tregs; ↑ plasma TNF-ɑ and IL-17A levels
Saresella (2009)	20 (Male: 14; Female: 6)	5 - 17	15 siblings, 20 controls (Male: 17; Female: 18)	3 - 16	↓ CD8^+^ EM; ↑ CD8^+^ Naive; ↑ pro-inflammatory and IL-10-producing immune cells
Yonk (1990)	25 (Male: 18; Female: 7)	3 - 31	25 siblings, 20 controls (Male: 33; Female: 22)	2 - 28	↓ number of T cells and B cells; ↓ percentage of CD4^+^ T cells; ↑ percentage of CD8 T cells

Representative studies were chosen based on the use of flow cytometry.

↓: decreased; ↑: increased.

The reduction in non-class switched memory B cells in children with autism compared to HC suggests a possible defect in B cell maturation. Currently, there is a rarity of studies directly examining the maturation process of B cells in children with autism, but perturbations in immunoglobulin production have been shown to be one of the most common immune irregularities observed in autistic children. Besides the presence of autoantibodies (8–10), it was also reported that children with autism express reduced levels of plasma IgG and IgM compared to HC, correlating with autism severity (70). Nevertheless, a subsequent study by the same group showed that there were no significant differences in memory B cell subpopulations, thus suggesting that the discrepancies in immunoglobulin production do not directly reflect B cell irregularities (71).

While we have identified several differences in the immunome of healthy children and children with autism, current studies regarding these subsets are inconclusive or conflicting, and are often over-generalized and scattered (**Table 2**). This is likely contributed by the small size of the studies (with many studies having less than 40 patients with autism), a wide range of ages at the time of blood sampling, and differences in experimental protocols. In addition, there is a strong lack of studies examining immune subsets in detail, and the published literature is not representative of the entire immunological profile. Therefore, we are unable to delineate the relationship between autism and CD4^+^ T cells, non-class-switched memory B cells, or NK cells, if any. We are also unable to evaluate any meaningful impact AUCB transfusion has on these subsets with regards to healthy levels. The many unknowns emphasize the need for a definitive study with a larger cohort of children with autism and appropriate age-matched controls to delineate the immunological changes in ASD with greater clarity.

### 4.1 Limitations of the study

One limitation of this study is the lack of more time points, both early and late (e.g. 1, 3, or 12 months post-transfusion). As a result, we were unable to observe if there were any changes or improvements in either the psychometric measures or the immunological aspects in the short-term before diminishing at 6-month post-transfusion, as well as any late-manifesting effects further along. Secondly, Panel B was specifically designed to delineate the B cell compartment; hence, it is lacking certain T cell markers and cytokines that would have allowed us to examine and cross-reference the CD4^+^ and CD8^+^ T cell subsets more in depth (e.g. CCR7, IFNɣ, TNFα) to identify the differences between patients with autism and HC. Our study has a small sample size and is exploratory in nature to discover the potential immunological changes that occur post-AUCB transfusion, which will form the basis of future validation studies. Lastly, as autism is a multi-system disorder involving both the immune and neural systems, we are unable to determine any causal effect directly linking immune and clinical changes, as we did not examine the contribution of the neural system.

In summary, we have described a holistic evaluation of the post-AUCB infused immunome using multi-parametric mass cytometry to delineate the effector and regulatory arms of the immune system. We did not find any evidence that AUCB infusion had any impact on the immunoregulatory Treg and Breg cells. Instead, we observed a decrease in CD4^+^ CM T cells and memory B cells that is attributable to AUCB infusion. Importantly, any immunological changes that were found did not translate to clinical benefits. Our study thus does not provide evidence that AUCB transfusion can be an effective immunomodulatory therapy, due to the lack of changes that demonstrate a clear dampening of immune effector response or enhancement of immunoregulatory response.

## Data Availability

The raw data supporting the conclusions of this article will be made available by the authors, without undue reservation.
