# Supplementary material for: Immune profiling shows limited systemic changes in children with autism after autologous cord blood transfusion
Source: Front Immunol. 2026 Jul 1;17:1738417. doi: 10.3389/fimmu.2026.1738417 (PMC13368730; doi:10.3389/fimmu.2026.1738417)
Supplement: Supplementary file 1 [file DataSheet1.pdf]

Supplementary Figure 1

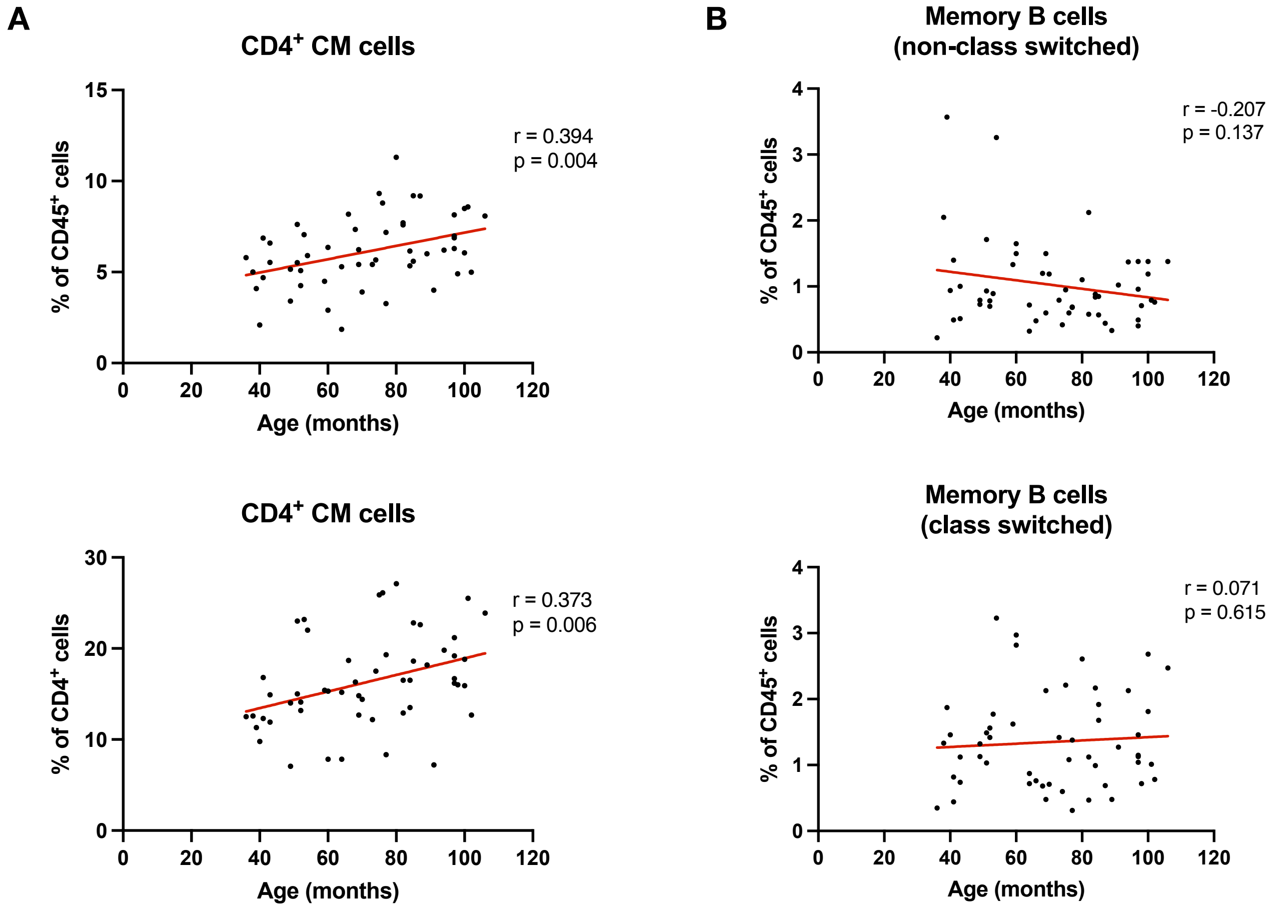

Supplementary Figure 1.
(A) Correlation between age and CD4+ CM cells as a percentage of total CD45+ cells (top) or as a percentage of CD4+ T cells (bottom). CD4+ CM cells were identified by supervised bivariate gating for CD56-CD14-CD3+CD8+CD45RA-CCR7+. **(B)** Correlation between age and non-class switched memory B cells (top) or class switched memory B cells (bottom) as a percentage of total CD45+ cells. Non-class switched memory B cells were identified by supervised bivariate gating for CD56-CD14-CD19+CD24-CD38-CD27+IgD+. Class switched memory B cells were identified by supervised bivariate gating for CD56-CD14-CD19+CD24-CD38-CD27+IgD-.

Supplementary Table 1

Panel A							
**Metal**	**Antibody**	**Isotype**	**Clone**	**Company**	**Catalogue Number**	**Lot Number**	**Concentration (µg/ml)**
**89**	**Barcode A**	mIgG1	HI30	Fluidigm	3089003B	25062603-03	10
**106**	**Barcode B**	mIgG1	HI30	Fluidigm	3106001B	25020672-13	10
**110**	**CD19**	mIgG1	HIB19	Biolegend	302202	B286428	12
**111**	**CD45RA**	Mouse IgG2b	HI100	Biolegend	304102	B373506	10
**112**	**CD14**	mIgG2a	Tuk4	Invitrogen	MA5-16956	X133495361	6
**113**	**Barcode C**	mIgG1	HI30	Fluidigm	3113001B	25073135-07	10
**114**	**CD56**	mIgG2b	NCAM16.2	BD	559043	9346558	8
**115**	**Barcode D**	mIgG1	HI30	Biolegend	304002	B283767	10
**116**	**CD8**	mIgG1	SK1	Biolegend	344702	B373506	5
**139**	**HLA-DR**	mIgG2a	L243	Biolegend	307602	B331589	6
**141**	**CD95**	mIgG1	DX2	Biolegend	305602	B383591	7.5
**142**	**CD27**	mIgG1	O323	Biolegend	302802	B402108	7.5
**143**	**CD3**	mIgG1	OKT3	Biolegend	317315	B238685	6
**144**	**IFN-γ**	mIgG1	B27	Biolegend	56502	B285057	0.6
**145**	**IL4**	mIgG1	8D4-8	Biolegend	500707	B237867	4
**146**	**Anti-FITC (TCRγδ)**	mIgG1	5A6.E9	Biolegend	MHG-D01	231107015	6
**147**	**PD-1**	mIgG1	EH12.2H7	Biolegend	329902	B365716	8
**148**	**CD4**	mIgG1	SK3	Biolegend	344602	B326479	5
**149**	**CD38**	mIgG1	A019D5	Biolegend	303502	B402099	4
**150**	**IL-22**	mIgG1	22URTI	Fluidigm	3150007B	23093795-05	7
**151**	**GATA3**	Rat IgG2b	TWAJ	eBioscience	14-9966-82	4349179	5
**152**	**TNF-α**	mIgG1	Mab11	Biolegend	502902	B223054	2
**153**	**CD25**	mIgG1	2A3	BD	340739	7355911	5
**154**	**IL-6**	Rat IgG1	MQ2-13A5	Fluidigm	3154011B	2801907	6.5
**155**	**CD152/CTLA4**	mIgG2a	BNI3	BD	555850	9066915	6
**156**	**CD28**	mIgG1	CD28.2	Biolegend	302923	B229977	6
**157**	**CXCR5**	Rat IgG2b	RF8B2	BD	552032	9024996	4
**158**	**CCR7**	mIgG2a	G043H7	Biolegend	353237	B331104	9
**159**	**CXCR3**	mIgG1	G025H7	Biolegend	353702	B394166	8
**160**	**CD161**	mIgG1	HP-3G10	Biolegend	339902	B260197	5
**161**	**CD138**	mIgG1	DL-101	Biolegend	352311	B372081	5
**162**	**CD154_CD40L**	mIgG1	24-31	Biolegend	310802	B349806	5
**163**	**TBX21/Tbet**	mIgG1	4B10	Biolegend	BE0100	3948/0612	5
**164**	**CD107a_PE**	mIgG1	H4A3	Biolegend	328608	B451789	10
**165**	**FoxP3**	Rat IgG2a	PCH101	eBioscience	14-4776-82	2678858	7
**166**	**IL-2**	Rat IgG2a	MQ1-17H12	Fluidigm	3166002B	2212633-020	5
**167**	**TCRα7.2**	Mouse IgG1	3C10	Biolegend	351702	B349475	7
**168**	**IgD**	mIgG2a	IA6-2	Biolegend	348202	B372086	3
**169**	**IL-17A**	mIgG1	BL168	Biolegend	512302	B186379	4
**170**	**iCOS**	Armenian Hamster IgG	C398.4A	Biolegend	313502	B402043	8
**171**	**TIGIT**	mIgG1	MBSA43	Invitrogen	16-9500-85	1965232	5
**172**	**Ki-67**	mIgG2	B56	Fluidigm	3172024B	23093944-12	5
**173**	**GranzymeB**	mIgG1	CLB-GB11	Abcam	ab103159	GR3413521-4	3.5
**174**	**CD69**	mIgG1	FN50	Biolegend	310902	B356123	5
**175**	**IL-10**	Rat IgG2a	JES3-19F1	Biolegend	506802	B286824	7
**176**	**CCR6/CD196**	mIgG2b	G034E3	Fluidigm	3176022A	162009	5
**191/193**	**DNA**	-	-	-	-	-	-
**195**	**Live/Dead**	-	-	-	-	-	-
**209**	**CD137/4-1BB**	mIgG1	4B4-1	Fluidigm	3209015B	25073260-16	5

Supplementary Table 1.
Antibodies used for mass cytometry (Panel A). The antibody clones, source, catalogue and lot numbers, concentration, and the mass cytometry channel used for their detection are listed here. Barcode A, B, C, and D are the different lanthanide metal-conjugated anti-human CD45 antibodies used for barcoding of the different PBMC samples prior to their staining with antibodies directed against cell surface antigens.

Supplementary Table 2

Panel B							
**Metal**	**Antibody**	**Isotype**	**Clone**	**Company**	**Catalogue Number**	**Lot Number**	**Concentration (µg/ml)**
**89**	**Barcode A**	mIgG1	HI30	Fluidigm	3089003B	24084510-13	10
**106**	**Barcode B**	mIgG1	HI30	Fluidigm	3106001B	25020672-13	10
**110**	**CD19**	mIgG1	HIB19	Biolegend	302202	B286428	12
**111**	**CD45RA**	Mouse IgG2b	HI100	Biolegend	304102	B373506	8
**112**	**CD14**	mIgG2a	Tuk4	Invitrogen	MA5-16956	X133495361	6
**113**	**Barcode C**	mIgG1	HI30	Fluidigm	3113001B	23062562-20	10
**114**	**CD56**	mIgG2b	NCAM16.2	BD	559043	9346558	8
**115**	**Barcode D**	mIgG1	HI30	Biolegend	304002	B283767	10
**116**	**CD8**	mIgG1	SK1	Biolegend	344702	B373506	5
**139**	**HLA-DR**	mIgG2a	L243	Biolegend	307602	B331589	6
**141**	**CD95**	mIgG1	DX2	Biolegend	305602	B383591	7.5
**142**	**IL6**	Rat IgG1	MQ2-13A5	Biolegend	501110	B206535	7
**143**	**CD3**	mIgG1	OKT3	Biolegend	317315	B238685	6
**144**	**CLA**	Rat IgM	HECA-452	Biolegend	321302	B269390	1.5
**145**	**CD11c**	mIgG1	Bu15	Biolegend	337221	B241039	8
**146**	**IgD**	mIgG2a	Ia6-2	Biolegend	348202	B167341	6
**147**	**PD-1**	mIgG1	EH12.2H7	Biolegend	329902	B365716	8
**148**	**CD4**	mIgG1	SK3	Biolegend	344602	B326479	5
**149**	**CD21**	mIgG1	Bu32	Biolegend	354902	B267500	5
**150**	**CD152/CTLA4**	mIgG2a	BN13	BD	555850	9066915	8
**151**	**IL-21**	mIgG1	3A3.N2	Biolegend	513009	B185588	5
**152**	**CD134/OX40**	mIgG1	Ber-ACT35	Biolegend	350015	B185598	18
**153**	**CD25**	mIgG1	2A3	BD	340739	7355911	5
**154**	**CD27**	mIgG1	O323	Biolegend	302802	B402108	6
**155**	**IgG**	mIgG1	G18-145	BD	555784	5234968	4
**156**	**CD38**	mIgG1	HIT2	Biolegend	303502	B285716	5
**157**	**CXCR5**	Rat IgG2b	RF8B2	BD	552032	9024996	5
**158**	**CD32B**	mIgG1	7.3	Abnova	MAB6930	251503	8
**159**	**CXCR3**	mIgG1	G025H7	Biolegend	353702	B394166	10
**160**	**CD154_CD40L**	mIgG1	24-31	Biolegend	310802	B349806	8
**161**	**CCR5**	mIgG1	T21/8	Abcam	ab110103	GR3264444-6	5
**162**	**CD138**	mIgG1	DL-101	Biolegend	352311	B372081	5
**163**	**TBX21/Tbet**	mIgG1	4B10	Biolegend	BE0100	3948/0612	5
**164**	**CX3CR1**	mIgG1	K0124E1	Biolegend	355702	B194773	8
**165**	**FoxP3**	Rat IgG2a	PCH101	eBioscience	14-4776-82	2678858	7
**166**	**IL-10**	Rat IgG1	JES3-9D7	Fluidigm	316600813	24094927-03	7
**167**	**CD24**	mIgG2a	ML5	Biolegend	311102	B280533	6
**168**	**CD252/OX40L**	mIgG1	11C3.1	Biolegend	326302	B215030	18
**169**	**IL-2**	Rat IgG2a	MQ1-17H12	Fluidigm	3166002B	2212633-020	7
**170**	**iCOS**	Armenian Hamster IgG	C398.4A	Biolegend	313502	B402043	8
**171**	**TIGIT**	mIgG1	MBSA43	Invitrogen	16-9500-85	1965232	5
**172**	**CD20**	mIgG2b	2H7	Biolegend	302302	B266249	8
**173**	**CD257/BAFF**	mIgG1	1D6	Biolegend	366502	B286549	7
**174**	**CD40**	mIgG1	5C3	Biolegend	334302	B201401	5
**175**	**IL-10**	Rat IgG2a	JES3-19F1	Biolegend	506802	B286824	7
**176**	**CD268/BAFFR**	mIgG1	11C1	Biolegend	316902	B277685	8
**191/193**	**DNA**	-	-	-	-	-	-
**195**	**Live/Dead**	-	-	-	-	-	-
**209**	**CD11b**	mIgG1	ICRF44	Fluidigm	3209003B	132007	6

Supplementary Table 2.
Antibodies used for mass cytometry (Panel B). The antibody clones, source, catalogue and lot numbers, concentration, and the mass cytometry channel used for their detection are listed here. Barcode A, B, C, and D are the different lanthanide metal-conjugated anti-human CD45 antibodies used for barcoding of the different PBMC samples prior to their staining with antibodies directed against cell surface antigens.
